# Clinical Outcomes Following Single vs. Multiple Vessel Living-Donor Kidney Transplantation: A Retrospective Comparison of 210 Patients

**DOI:** 10.3389/fsurg.2021.693021

**Published:** 2021-06-14

**Authors:** Leonardo E. Garcia, Natalia Parra, Jeffrey J. Gaynor, Lauren Baker, Giselle Guerra, Gaetano Ciancio

**Affiliations:** ^1^Jackson Memorial Hospital, University of Miami Miller School of Medicine, Miami, FL, United States; ^2^Departments of Surgery and Urology, University of Miami Miller School of Medicine, Miami, FL, United States; ^3^Division of Nephrology, Department of Medicine, Miami Transplant Institute, University of Miami Miller School of Medicine, Miami, FL, United States

**Keywords:** living-donor kidney transplantation, multiple donor arteries, vascular reconstruction, clinical outcomes, retrospective cohort analysis

## Abstract

**Background:** The use of living-donor kidney allografts with multiple vessels continues to rise in order to increase the donor pool. This requires surgeons to pursue vascular reconstructions more often, which has previously been associated with a higher risk of developing early post-transplant complications. We therefore wanted to investigate the prognostic role of using living-donor renal allografts with a single artery (SA) vs. multiple arteries (MA) at the time of transplant.

**Methods:** We retrospectively analyzed a cohort of 210 consecutive living-donor kidney transplants performed between January, 2008 and March, 2019, and compared the incidence of developing postoperative complications and other clinical outcomes between SA vs. MA recipients.

**Results:** No differences were observed between SA (*N* = 161) and MA (*N* = 49) kidneys in terms of the incidence of developing a postoperative (or surgical) complication, a urologic complication, hospital length of stay, delayed graft function, estimated glomerular filtration rate at 3 or 12 mo post-transplant, and graft survival.

**Conclusions:** The use of live-kidney allografts with MA requiring vascular reconstruction shows excellent clinical outcomes and does not increase the risk of developing postoperative complications or other adverse outcomes when compared with SA renal allografts.

## Introduction

Kidney transplantation has long been established as the optimal therapy for patients with end-stage renal disease (ESRD). Living kidney donation has increased substantially in the attempt to meet current demands, leading to a surge in transplantation of kidney allografts with multiple arteries. These allografts often require complex back-table reconstruction prior to transplantation, which has been linked with poorer post-transplant outcomes in their recipients, when compared with single artery kidney allografts (1–5). The aim of this study was to describe the short-term clinical outcomes of living-donor kidney transplant recipients of multiple vessel allografts requiring vascular reconstruction, and evaluate other baseline factors that may influence short-term postoperative outcomes. Specifically, we were interested in evaluating the incidence of post-operative (surgical) complications that occurred during the first 30 days (12 months) post-transplant with special interest in the following urological complications: renal allograft thrombosis, peri-renal hematoma, lymphocele, ureteral leak, ureteral stenosis, and vesicoureteral reflux. Summarized below are the results of this observational study.

## Materials and Methods

**Figure 1:**
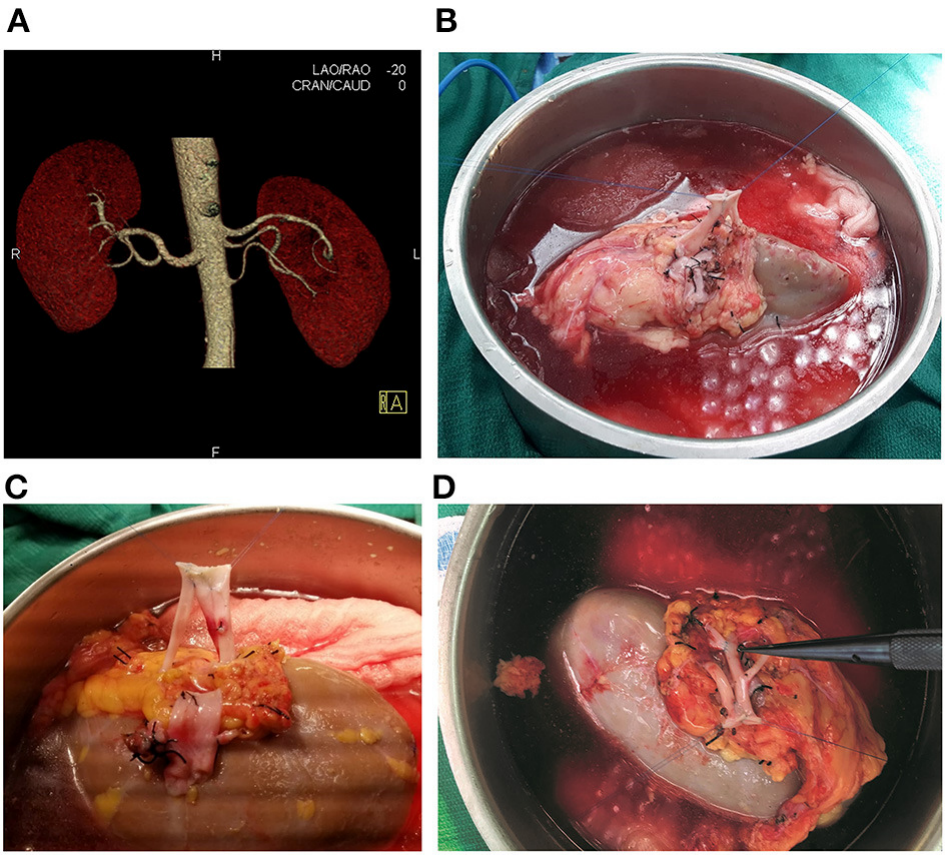
**(A)** Computed Tomography Angiography showing the left kidney with three renal arteries. **(B)** Living donor kidney with two arteries, conjoined side-to side with 8-0 Prolene. **(C)** Living donor kidney with three arteries, with all three renal arteries conjoined side-to side with 8-0 Prolene. **(D)** Living donor kidney with two main renal arteries and an upper pole accessory renal artery. The two renal arteries were anastomosed side-to-side with 7-0 Prolene, and the accessory upper pole artery was anastomosed end-to-side into one of the main renal arteries with 8-0 Prolene suture inside the renal hilum.

Between February 2008 and March 2019, 210 patients underwent consecutive living-donor kidney transplantation by a single surgeon (G.C.) at our institution (of note, this highly experienced, single surgeon has been performing kidney transplants since 1993 and has been a faculty member at the Miami Transplant Institute since 1995). A retrospective review of these 210 consecutively transplanted patients was approved by the University of Miami Institutional Review Board and follows the ethical principles (as revised in 2013) of the Helsinki Declaration. All patients gave written informed consent prior to enrollment. The donor vascular renal anatomy was evaluated with computed tomography angiography (Figure 1A).

In living-donor kidney transplantation, our strategy is to always use the donor kidney (left or right) considered to be the less favorable kidney, allowing the donor to retain the more favorable kidney. In cases where both the left and right donor kidneys are equally favorable, the choice of which donor kidney to use for transplant is then based on the ease in performing the donor nephrectomy, minimizing surgical risks to both the donor and recipient. For instance, a right donor kidney with a short renal vein would favor using the left kidney for transplant; however, the presence of multiple renal arteries in one kidney might favor the removal of the other kidney.

### Surgical Technique

Following hand-assisted laparoscopic extraction of the donor kidney, standard benching preparation was performed, and the graft was flushed with cold Histidine-tryptophan-ketoglutarate until the effluent was clear. The renal arteries and veins were dissected from the surrounding perivascular lymphatics and fat, and the side branches were ligated. The ureter with its blood supply and the periureteric tissue were preserved, and all remaining redundant perinephric fat was trimmed. In the instance of multiple renal vessels, different back-table techniques were adopted to reconstruct the renal arteries or veins depending on their lengths and calibers.

**Figure 2:**
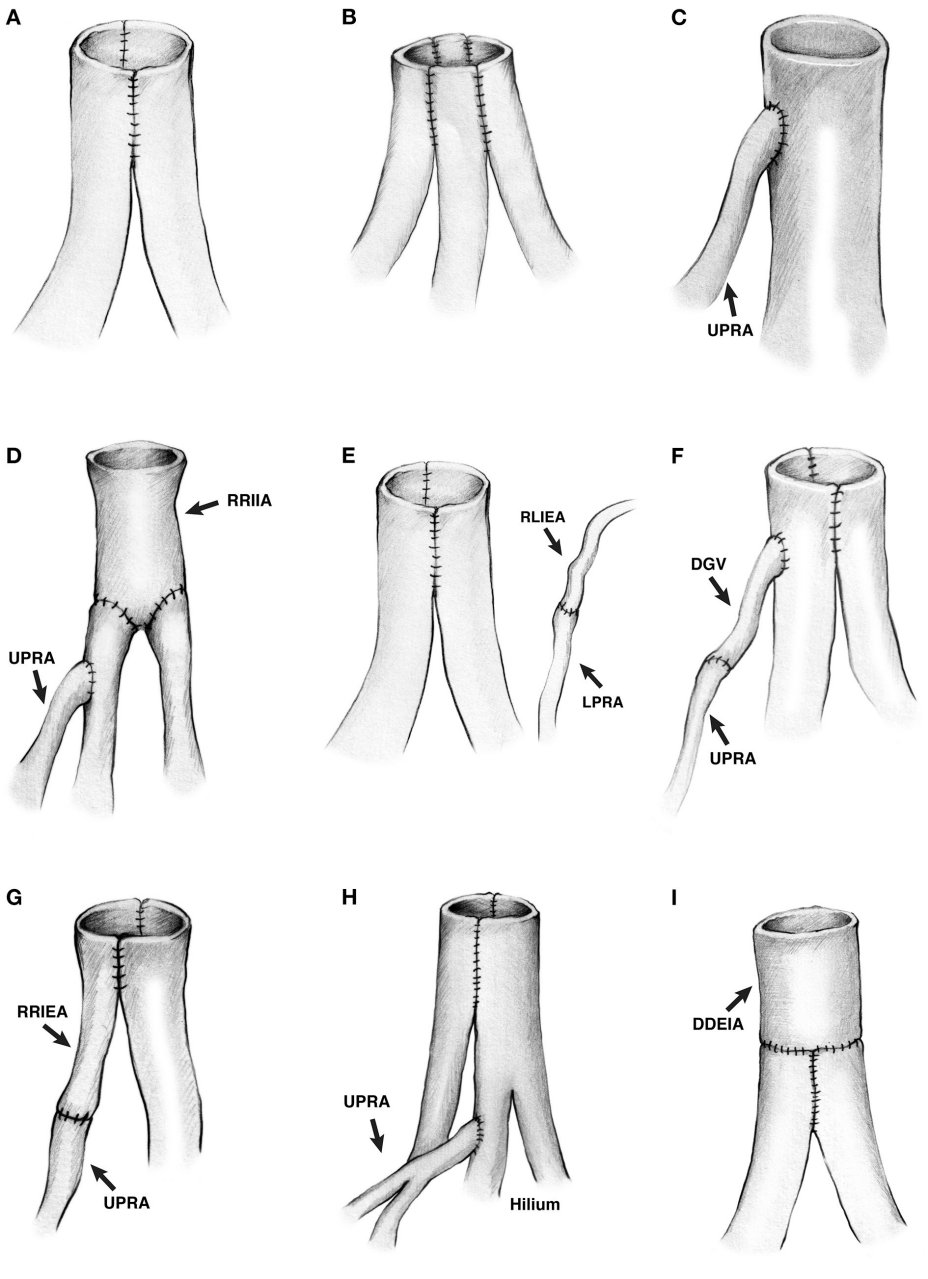
**(A–H)** Drawing of the different living donor renal artery reconstructions. **(A)** Two renal arteries conjoined side-to-side. **(B)** Three renal arteries conjoined side-to-side. **(C)** A small upper pole renal artery anastomosed end-to-side into the major renal artery. **(D)** A segment of recipient right internal iliac artery was anastomosed end-to-end to two renal arteries, and a small upper pole renal artery was anastomosed end-to-side into the upper renal artery. **(E)** Two renal arteries conjoined side-to-side, and a lower pole renal artery was anastomosed end-to-end to the recipient's left inferior epigastric artery. **(F)** Two renal arteries conjoined side-to-side, and an upper pole renal artery was anastomosed end-to-side into the upper major renal artery using the donor gonadal vein as an interposition graft to increase the length of the upper pole renal artery. **(G)** An upper pole renal artery was anastomosed side-to-side to the renal artery using a recipient right inferior epigastric artery as interposition graft to increase the length of the branch. **(H)** Two renal arteries conjoined side-to-side, and a short upper pole renal artery was anastomosed end-to-side into one of the branches of the upper renal artery. The anastomosed was inside the hilum. **(I)** Two renal arteries conjoined side-to-side, then anastomosed end-to-end to a deceased donor external iliac artery to increase the length. Both renal arteries were short. UPRA indicates upper pole renal artery; RRIIA, right recipient internal iliac artery; LPRA, lower pole renal artery; RLIEA, recipient left inferior epigastric artery; DGV, donor gonadal vein, RRIEA, recipient right inferior epigastric artery; DDEIA, deceased donor external iliac artery.

Double-barrel side-to-side vascular reconstruction was most frequently used (*N* = 36, 33/36 with two renal arteries, 3/36 with three renal arteries; Figures 1B,C, 2A,B), followed by end-to-side anastomosis of a shorter branch into the main renal artery (*N* = 6, upper pole branch in 4 cases, lower pole branch in 2 cases, 6/6 with two renal arteries) (Figure 2C). In the 3/36 cases with three renal arteries, all three arteries were conjoined side-to-side. In one additional donor kidney with three renal arteries, one very small artery (1 mm in diameter) was tied off, and the other two renal arteries were conjoined side-to-side.

More complex vascular reconstructions were performed in the remaining six donor kidneys having two or three renal arteries. In the 1st case with three renal arteries (Figure 2D), one short artery was anastomosed end-to-side into one of two main renal arteries. The two main renal arteries had been anastomosed end-to-end into a segment of the recipient's internal iliac artery (on the back table). In the 2nd case with three renal arteries (Figure 2E), two arteries were conjoined side-to-side, and the lower pole artery was anastomosed end-to-end (after reperfusion) into the recipient's left inferior epigastric artery. In this particular case, it was simpler to perform two arterial anastomoses, avoiding a more cumbersome attempt to combine all three arteries together (on the back table). In the 3rd case with three donor arteries (Figure 2F), the upper pole branch was anastomosed end-to-end into the donor gonadal vein (on the back table), which was then anastomosed end-to-side into one of the two main renal arteries. The two main renal arteries had been conjoined side-to-side. In the 4th case with two renal arteries (Figure 2G), the upper pole branch was anastomosed end-to-end into a segment of the recipient's inferior epigastric artery (on the back table), which was then conjoined side-to-side with the main renal artery. In the 5th case with three donor arteries (Figures 1D, 2H), the upper pole branch was anastomosed end-to-side into a branch of one of the two main renal arteries. The two main renal arteries had been conjoined side-to-side. In the 6th case with two donor arteries (Figure 2I), the two donor arteries were first conjoined side-to-side; however, since they were short, they were then anastomosed end-to-end into a segment of a deceased donor's external iliac artery. In summary, with the exception of one case in which two arterial anastomoses were performed (Figure 2E), all of the other single and multiple vessel transplants were performed using a single arterial anastomosis.

Once reperfusion of the graft was achieved, mobilization of the bladder with subsequent extravesical ureteroneocystostomy was carried out using two running 6-0 polydioxanone sutures. Finally, the detrusor muscle was closed over the anastomosis to create an anti-reflux tunnel with interrupted 4-0 PDS sutures (6).

Of note, routine placement of a double-J ureteral stent at the time of transplant was not performed in this series; the decision to place a stent was made by the transplant surgeon (G.C.).

After surgery and prior to hospital discharge, patients had daily measurements of serum creatinine, BUN, and electrolytes, a complete blood count, and Doppler and gray scale renal ultrasonography as baseline.

### Immunosuppression

All recipients received immunosuppressant therapy according to protocols at our center, with induction consisting of intravenous antithymocyte globulin 1 mg/kg, basiliximab 20 mg, and methylprednisolone 500 mg administered intraoperatively before organ reperfusion. Maintenance immunosuppression included a steroid-free regimen consisting of tacrolimus and mycophenolate mofetil, starting on postoperative day 1 (7).

### Data Preparation

At the Miami Transplant Institute (MTI)/Jackson Memorial Hospital (JMH), all patients are prospectively followed. When a kidney transplant patient is seen either as an inpatient in the hospital or as an outpatient in the transplant clinic, both the attending physicians (surgeon and transplant nephrologist) and transplant-dedicated nurses record detailed records of all medical history, procedures performed, drugs given, and clinical status of the patient. This information is updated daily while the patient is in the hospital (at JMH). These documented records are kept within 2 distinct databases at our center: Cerner (the JMH patient database), and OTTR (the MTI database). The first 4 co-authors of this manuscript (LEG, NP, JJG, and LB) along with the senior author (GC) had retrospectively organized the relevant patient data into a clinical research EXCEL file. Any checks of the information recorded into this EXCEL file were made via re-review of the original information recorded in Cerner and OTTR. So, while all clinical outcomes were initially recorded (and thus, permanently stored) in the JMH and MTI databases by the attending physicians (and nurses) who saw the patients while hospitalized or as an outpatient in the transplant clinic, the co-authors of this manuscript reviewed the relevant clinical data on each patient and then transcribed it into an EXCEL file. The statistical analysis was then performed using data contained in this EXCEL file.

### Statistical Analysis

Data from patients were retrospectively organized according to the number of renal vessels or anomalies reported in the donor operative report and were entered into an EXCEL file. Analyzed baseline variables included date of transplant, recipient age, recipient gender, recipient race/ethnicity, recipient BMI, recipient pretransplant history of diabetes mellitus (no/yes), kidney retransplant status (no/yes), donor kidney location (left or right), number of donor arteries, number of donor veins, need for vascular reconstruction, living donor type (related/unrelated), double-J ureteral stent insertion (no/yes), operative time, cold ischemic time, and warm ischemic time. The primary outcome variable was the development of a post-operative (or surgical) complication during the first 30 days (12 months) post-transplant. Secondary outcomes included length of hospital stay, development of delayed graft function (DGF) (requirement for dialysis during the first post-operative week, no/yes), postoperative creatinine, development of a urologic complication during the first 12 months post-transplant, and graft loss (return to permanent dialysis or death). Estimated glomerular filtration rate (eGFR) was calculated using the “Chronic Kidney Disease Epidemiology Collaboration Equation.” Percentages of patients having selected baseline characteristics were determined as well as means and standard errors for baseline continuous variables. Pearson (uncorrected) chi-square and ordinary (2-sided) *t*-tests were used to test the associations of categorical and continuous variables with the number of donor renal arteries (1 vs. >2), respectively. Stepwise linear (logistic) regression was performed to determine multivariable baseline predictors of the likelihood of requiring vascular reconstruction at the time of transplant (development of a post-operative or surgical complication). Time-to-event variables (e.g., graft survival) were compared using the Kaplan-Meier technique and log-rank tests. *P*-values ≤ 0.05 were considered to be statistically significant.

The date of last follow-up for this study was March 20, 2020. Median follow-up among 195 patients who were alive with a functioning graft as of the last follow-up date was 26.2 (range: 12.0–128.4) months post-transplant. Of note, the minimum follow-up for all patients who were alive with a functioning graft was 12 months post-transplant.

## Results

Distributions of demographic characteristics and post-operative outcomes appear in Table 1A. Mean recipient age (±SE) was 49.4 ± 1.1 years; 64.3% (135/210) were male. Blacks and Hispanics comprised 19.0% (40/210) and 36.2% (76/210) of the transplant recipients, respectively. The majority of transplant recipients, 96.7% (203/210), received a primary kidney transplant; only 3.3% (7/210) were retransplant cases. The percentage of recipients who received a right donor kidney was 10.0% (21/210); 90.0% (189/210) received a left donor kidney. The percentage who received a kidney with 1, 2, and 3 donor arteries was 76.7% (161/210), 19.5% (41/210), and 3.8% (8/210), respectively. Two patients (1.0%) received a donor kidney with two renal veins – the donor kidney in one of these two patients also had three renal arteries, while the other had just one renal artery. Of note, at the time of transplant a double-J ureteral stent was placed in only 8.6% (18/210) of the patients. Also of note, the percentage receiving a kidney with >2 donor arteries was similar between those who received a left vs. right donor kidney, 23.8% (45/189) vs. 19.0% (4/21) (*P* = 0.62).

**Table 1A:** Distributions of selected baseline variables and outcome variables (*N* = 210).

	Mean ± SE if continuous (Geometric Mean */ SE for Variables with Skewed Distributions)
**Baseline variable**	**Percentage with characteristic if categorical**
**DOT**	
<2015	38.6% (81/210)
≥2015	61.4% (129/210)
**Recipient Age (yr)**	49.4 ± 1.1 (*N* = 210) (Median = 51.8, Range: 4–80)
**Recipient age (yr)**	
<18	3.3% (7/210)
≥18, <50	42.4% (89/210)
≥50	54.3% (114/210)
**Recipient gender**	
Female	35.7% (75/210)
Male	64.3% (135/210)
**Recipient race/ethnicity**	
Black (non-Hispanic)	19.0% (40/210)
Hispanic	36.2% (76/210)
White (non-Hispanic)	42.4% (89/210)
Other	2.4% (5/210)
Recipient BMI (kg/m^2^)	26.4 ± 0.4 (*N* = 208) (Median = 26.2, Range: 13.9–42.4)
**Recipient pretransplant diabetes mellitus**	
No	79.5% (167/210)
Yes	20.5% (43/210)
**Retransplant**	
No	96.7% (203/210)
Yes	3.3% (7/210)
**Donor type**	
Living related	53.8% (113/210)
Living unrelated	46.2% (97/210)
**Kidney**	
Right	10.0% (21/210)
Left	90.0% (189/210)
**Number of donor arteries**	
1	76.7% (161/210)
2	19.5% (41/210)
3	3.8% (8/210)
**Number of donor veins**	
1	99.0% (208/210)
2	1.0% (2/210)
**Vascular reconstruction**	
No	73.3% (154/210)
Yes	26.7% (56/210)
**Double-J ureteral stent placed**	
No	91.4% (192/210)
Yes	8.6% (18/210)
**CIT (h)**	0.86 ± 0.03 (*N* = 209) (Median = 0.75, Range: 0.17–3.33)
**WIT (min)**	30.40 */ 1.02 (*N* = 209) (Median = 28, Range: 16–117)
**EBL (ml)**	36.2 */ 1.06 (*N* = 210) (Median = 30.0, Range: 10–780)
**Operative Time (h)**	4.17 ± 0.08 (*N* = 210) (Median = 4.0, Range: 1.0–8.4)
**Length of Hospital Stay (days)**	4.40 */ 1.03 (*N* = 210) (Median = 4, Range: 2–67)
**Developed DGF**	
No	100.0% (210/210)
Yes	0.0% (0/210)
**Developed a post-operative (or surgical) complication within 30 days (12 months) post-transplant^a^**	
No	96.2% (202/210)
Yes	3.8% (8/210)
**Developed a urologic complication within 12 months post-transplant^a^**	
No	98.6% (207/210)
Yes	1.4% (3/210)
**eGFR at 3 mo post-tx (ml/min/1.73m**^**2**^**)**	77.1 ± 1.7 (*N* = 205) (Median = 73.0, Range: 32.5–206.7)
**eGFR at 6 mo post-tx (ml/min/1.73m**^**2**^**)**	76.1 ± 1.8 (*N* = 200) (Median = 75.0, Range: 8.3–190.3)
**eGFR at 12 mo post-tx (ml/min/1.73m**^**2**^**)**	75.5 ± 1.7 (*N* = 198) (Median = 73.2, Range: 15.6–174.8)
**eGFR at 36 mo post-tx (ml/min/1.73m**^**2**^**)**	71.3 ± 2.3 (*N* = 93) (Median = 69.9, Range: 12.0–148.1)
**eGFR at 60 mo post-tx (ml/min/1.73m**^**2**^**)**	64.9 ± 3.4 (*N* = 56) (Median = 68.3, Range: 6.2–113.8)
**Graft failure (i.e., return to permanent dialysis or retransplanted) (as of the last follow-up date)^b^**	
No	97.1% (204/210)
Yes	2.9% (6/210)
**Death with a functioning graft (as of the last follow-up date)^b^**	
No	95.7% (201/210)
Yes	4.3% (9/210)
**Graft loss (death uncensored) (as of the last follow-up date)^b^**	
No	92.9% (195/210)
Yes	7.1% (15/210)

a *Among the eight patients who developed a post-operative (or surgical) complication during the first 30 days (12 months) post-transplant, the following complications were observed: acute respiratory distress syndrome (ARDS) (N = 1), wound infection (N = 1), wound infection/necrosis (N = 1), c. difficile colitis/sepsis (N = 1), diverticulitis (N = 1), bladder leak (N = 1), ureteral stricture (N = 1), and possible ureteral leak (N = 1)*.

b *The date of last follow-up for this study was March 20, 2020. Median follow-up among 195 patients who were alive with a functioning graft as of the last follow-up date was 26.2 (range: 12.0–128.4) months post-transplant. The six causes and times-to-graft failure (return to permanent dialysis) were as follows (listed chronologically by time to graft failure): acute AMR at 5.4 months, acute T-cell mediated rejection at 41.8 months, MPGN recurrence at 58.0 months, CAI at 67.7 months, CAI at 68.2 months, and acute AMR/non-adherence at 86.7 months post-transplant. The nine causes of death with a functioning graft and times-to-death were as follows: cardiovascular event in six patients (at 3.3, 5.2, 7.9, 12.9, 59.9, and 69.6 months post-transplant), and infection/sepsis in three patients (at 0.8, 12.2, and 17.4 months post-transplant)*.

There were three reasons for using the right donor kidney for transplant: (#1) the left donor kidney had multiple arteries, (#2) the right donor kidney was either smaller or had decreased function (or both), and (#3) the right donor kidney had one or more cysts (or kidney stones). Among the 21 recipients of a right donor kidney, reasons for using the right donor kidney were as follows: #1 only (*N* = 7), #2 only (*N* = 4), #3 only (*N* = 8), #1 combined with #3 (*N* = 1), and #2 combined with #3 (*N* = 1).

Also of note, among the seven kidney retransplant cases, six were 1st retransplants, and one was a 2nd retransplant. In five cases, the first kidney was placed extraperitoneally in the right iliac fossa, and the retransplanted kidney was placed extraperitoneally in the left iliac fossa. For the 2nd retransplant case, the first two kidneys had been placed extraperitoneally in the right and left iliac fossae, respectively, and the third kidney was placed intraperitoneally on the left side. Finally, in one patient who received a simultaneous pancreas-kidney transplant as the 1st transplant, the first kidney graft was placed intraperitoneally in the left iliac fossa. In performing the kidney retransplant for this case, the primary kidney graft was explanted, and the 2nd kidney was placed intraperitoneally in the same iliac fossa (left side). Thus, in only this latter case (1/7) was the same iliac fossa used in placing the retransplanted kidney.

Overall, the percentage of recipients who required vascular reconstruction was 26.7% (56/210); 100% (49/49) of the patients who received a donor kidney with >2 renal arteries had vascular reconstruction. Among the seven recipients of allografts having one renal artery but still requiring vascular reconstruction, three patients who received a right donor kidney had a short donor vein that required extension (in one instance, one of the donor's gonadal veins was laparoscopically harvested along with the kidney allograft and was used as an interposition graft to elongate the short donor renal vein). In the other two cases, an interposition graft was used in a similar fashion from a deceased donor common iliac vein. Among the four other cases with a single renal artery that required vascular reconstruction, two patients required aneurysm repair, one patient received a right donor kidney with two small renal veins that were conjoined, and 1 7-year old patient required thrombectomy of the renal vein which was observed intra-operatively as a web-like membrane within the lumen, most likely representing a neonatal thrombosis.

None of the 210 patients (0.0%) developed DGF. Eight patients (3.8%) developed a post-operative (or surgical) complication during the first 30 days (12 months) post-transplant, including: bladder leak at 2 days post-transplant (*N* = 1), c. difficile colitis/sepsis at 4 days post-transplant (*N* = 1), diverticulitis at 4 days post-transplant (*N* = 1), a suspected ureteral leak at 4 days post-transplant (*N* = 1), acute respiratory distress syndrome (ARDS) at 6 days post-transplant (*N* = 1), wound infection/necrosis at 2 weeks post-transplant (*N* = 1), wound infection at 6.6 months post-transplant (*N* = 1), and ureteral stricture at 9.0 months post-transplant (*N* = 1). The patient who developed c. difficile colitis/sepsis died of that infection (with a functioning graft) at 0.8 months post-transplant. None of the other seven patients who developed a post-operative (or surgical) complication within 30 days (12 months) post-transplant experienced graft loss during subsequent follow-up. The Clavien-Dindo classification grades of these 8 post-operative complications were: III, V, III, III, IV, III, II, and III, respectively.

Among the three patients (1.4%) who developed a urologic complication, a bladder leak observed in one patient was caused by traumatic injury during insertion of a Jackson-Pratt drain. This patient's bladder leak was surgically repaired. A ureteral stricture that developed in another patient was successfully treated with a percutaneous nephro-ureteral stent placement. Similarly, a ureteral leak that was suspected to have developed in a third patient (i.e., it was never actually found) was successfully treated with a percutaneous nephro-ureteral stent placement. One of the three patients who developed a urologic complication (the patient who developed a ureteral stricture) had a ureteral stent placed at the time of transplant. Of note, none of these three patients suffered any long-term consequences of their urological complications.

As of the last follow-up date, six patients (2.9%) have developed graft failure (i.e., returned to permanent dialysis). The six causes and times-to-graft failure were as follows (listed chronologically by time-to-graft failure): Acute antibody mediated rejection (AMR) at 5.4 months, acute T-cell mediated rejection at 41.8 months, MPGN recurrence at 58.0 months, chronic allograft injury (CAI) at 67.7 months, CAI at 68.2 months, and acute AMR/Nonadherence at 86.7 months post-transplant. Nine patients have died with a functioning graft. The nine causes of death with a functioning graft and corresponding times-to-death were as follows: cardiovascular event in six patients (at 3.3, 5.2, 7.9, 12.9, 59.9, and 69.6 months post-transplant), and infection/sepsis in three patients (at 0.8, 12.2, and 17.4 months post-transplant).

Associations of selected baseline and clinical outcome variables with the number of donor renal arteries (1 vs. >2) are shown in Table 1B. Number of donor arteries was significantly associated with three other variables: (i) the percentage who required vascular reconstruction was significantly higher among recipients of a donor kidney with >2 renal arteries, 100.0% (49/49) vs. 4.3% (7/161) among recipients of a single artery kidney (*P* < 0.000001); (ii) mean cold ischemia time was significantly longer among recipients of a donor kidney with >2 renal arteries, 1.31 ± 0.07 vs. 0.72 ± 0.02 h among recipients of a single artery kidney (*P* < 0.000001); and (iii) mean operative time was significantly longer among of recipients of a donor kidney with >2 renal arteries, 5.03 ± 0.14 vs. 3.90 ± 0.08 h among recipients of single artery kidneys (*P* < 0.000001). There was no notable association of the number of donor arteries with any of the outcome variables, including development of a post-operative (or surgical) complication, mean eGFR at various time points, and graft survival (Table 1B).

**Table 1B:** Associations of selected baseline and clinical outcome variables with number of donor arteries (1 vs. ≥2).

	**Number of donor arteries**		
**Baseline variable**	**1 (*N* = 161)**	**≥2 (*N* = 49)**	***P*-value**
DOT ≥ 2015	60.2% (97/161)	65.3% (32/49)	0.52
Mean recipient age (yr)	50.0 ± 1.3 (*N* = 161)	47.3 ± 2.2 (*N* = 49)	0.33
Male recipient	63.4% (102/161)	67.3% (33/49)	0.61
Black (non-hispanic) recipient	21.1% (34/161)	12.2% (6/49)	0.17
Hispanic recipient	34.8% (56/161)	40.8% (20/49)	0.44
Mean recipient BMI (kg/m^2^)	26.2 ± 0.4 (*N* = 160)	27.3 ± 0.8 (*N* = 48)	0.24
Recipient pretransplant DM	19.3% (31/161)	24.5% (12/49)	0.43
Retransplant (kidney)	2.5% (4/161)	6.1% (3/49)	0.21
LU (vs. LD) kidney recipient	45.3% (73/161)	49.0% (24/49)	0.65
Right kidney	10.6% (17/161)	8.2% (4/49)	0.62
2 (vs. only 1) donor vein(s)	0.6% (1/161)	2.0% (1/49)	0.37
Vascular reconstruction^a^	4.3% (7/161)	100.0% (49/49)	<0.000001
Double-J ureteral stent placed	9.3% (15/161)	6.1% (3/49)	0.48
Mean CIT (h)	0.72 ± 0.02 (*N* = 160)	1.31 ± 0.07 (*N* = 49)	<0.000001
Mean WIT (min)	29.8 */ 1.03 (*N* = 160)	32.4 */ 1.05 (*N* = 49)	0.14
Mean estimated blood loss (cc)	35.8 */ 1.07 (*N* = 161)	37.8 */ 1.12 (*N* = 49)	0.69
Mean operative time (h)	3.90 ± 0.08 (*N* = 161)	5.03 ± 0.14 (*N* = 49)	<0.000001
Mean length of hospital stay (days)	4.31 */ 1.03 (*N* = 161)	4.72 */ 1.08 (*N* = 49)	0.30
Developed DGF	0.0% (0/161)	0.0% (0/49)	1.00
Developed a post-operative complication	3.7% (6/161)	4.1% (2/49)	0.91
Developed a urologic complication	1.9% (3/161)	0.0% (0/49)	0.34
Mean eGFR (ml/min × 1.73 m^2^) at 3 mo	78.1 ± 2.1 (*N* = 157)	74.0 ± 2.9 (*N* = 48)	0.26
Mean eGFR (ml/min × 1.73 m^2^) at 12 mo	76.7 ± 2.0 (*N* = 154)	71.6 ± 3.2 (*N* = 44)	0.22
Developed graft failure	3.1% (5/161)	2.0% (1/49)	0.75
Death with a functioning graft	3.7% (6/161)	6.1% (3/49)	0.38
Developed (death uncensored) graft loss	6.8% (11/161)	8.2% (4/49)	0.64

a *Of note, seven patients that had only one donor artery still required vascular reconstruction: three patients who received a right donor kidney had a short donor vein that required extension; two patients required aneurysm repair; one patient (who received a right donor kidney) had two donor veins that were conjoined; and one patient required vascular reconstruction due to a thrombectomy of the renal vein that occurred at the time of transplant*.

Stepwise linear regression to determine multivariable predictors of which patients required vascular reconstruction at the time of transplant yielded two significant predictors: receiving a donor kidney with >2 renal arteries (*P* < 0.000001) and receiving a right donor kidney (*P* = 0.00002). Once these two variables were selected, no other variables contained additional predictive value (*P* > 0.05). In fact, none of the other baseline variables were associated in univariable analysis with the requirement of vascular reconstruction (*P* > 0.10).

Finally, stepwise logistic regression to determine multivariable predictors of developing a post-operative (or surgical) complication (eight events) yielded no significant predictors (*P* > 0.10). Of note, the observed percentage who developed a post-operative (or surgical) complication for: (i) recipients of a donor kidney with 1 vs. 2–3 renal arteries was 3.7% (6/161) vs. 4.1% (2/49), respectively (*P* = 0.91), (ii) recipients of a left vs. right donor kidney was 4.2% (8/189) vs. 0.0% (0/21), respectively (*P* = 0.34), and (iii) recipients who did not require vs. required vascular reconstruction was 3.2% (5/154) vs. 5.4% (3/56), respectively (*P* = 0.48).

## Discussion

Living-donor kidney transplantation has evolved in an attempt to keep up with the increasing demand of patients requiring transplantation (8). Donor vascular anomalies were initially viewed as a contraindication, as they posed significant technical challenges in open nephrectomy transplant surgery, as well as with subsequent laparoscopic techniques. However, the use of difficult donor allografts has rapidly increased, showing it to be a crucial component of the donor pool (9). Incidence of donor kidneys with multiple arteries being used in living-donor kidney transplantation has been described between 18 and 30% in previous series (10). These allografts with multiple vessels were initially associated with high rates of graft thrombosis, renal artery stenosis, and renovascular hypertension. Moreover, longer operative times were believed to add unnecessary risk to the donor, while potential vascular complications threatened the outcome of the allograft for the recipient (11–13).

A retrospective study by Ghazanfar et al. (13) on 201 living-donor kidney transplants found an 8.9 vs. 2.8% incidence of vascular complications in patients with and without multiple renal arteries, respectively; specifically, a higher incidence of renal artery stenosis was detected in that series. Similarly, Paramesh et al. (14) compared long-term graft function and survival of kidneys with single arteries (SA) vs. multiple arteries (MA) over a 10-year period, reporting an estimated graft survival at 1, 3, and 5 years of 94.4, 90.6, and 86% for the SA group (*N* = 218) vs. 89.6, 83.2, and 71.8% for the MA group (*N* = 60). Furthermore, there was a higher percentage of graft loss from chronic allograft nephropathy in the MA group than in the SA group, and the presence of multiple arteries was an independent risk factor for both acute rejection and graft loss, validating their conclusion that laparoscopic procurement of living-donor kidneys with single arteries yields lower risks (14). Conversely, Hsu et al. (15) retrospectively analyzed 353 patients undergoing living-donor kidney transplantation, identifying 277 kidney allografts with a single renal artery and 76 with multiple renal arteries. Although total operative time and allograft warm ischemia time differed between the two groups, clinical outcomes were not significantly different between the two groups (15). Additional studies have demonstrated no significantly unfavorable impact of multiple renal arteries or prolonged warm ischemia time on recipient renal function at 1 and 5-years post-transplant or graft survival (16). Results of a large meta-analysis (17), which included the first three studies mentioned above, showed small but nonetheless significant differences in the incidence of post-operative vascular and urologic complications in favor of SA vs. MA. However, despite these reported small differences in early post-operative outcomes favoring SA vs. MA, graft and patient survival at 5 years post-transplant were comparable. Thus, we still believe that the overall consensus (17–20) supports the notion that benefits from using multiple vessel kidneys outweigh any inevitable small increases in early post-operative complication rates, operative times, and/or warm ischemia times.

In our series of 210 patients who underwent living-donor kidney transplantation, we identified 56 (26.7%) patients requiring vascular reconstruction of either the renal arteries, renal veins, or both. Our results were in line with previous literature in which neither short nor long-term recipient outcomes were affected by initial allograft anatomy. This highlights the fact that a meticulous technique triumphs over vessel multiplicity, as the good results achieved in our study appear to be due, at least in part, to the careful vascular reconstructions that were performed. Clearly, the surgeon's experience in handling multiple renal vessels plays a role in determining the incidence of post-operative/surgical complications that may develop following living-donor kidney transplantation.

Of special interest is the use of ureteral stents in living-donor kidney recipients at the time of transplant. Double-J stents have been described as crucial in preventing mechanical ureteral complications in renal transplantation, with definite indications for patients with neurogenic bladder or previous bladder surgery; however, their use remains controversial due to a potentially increased risk of developing hematuria and urinary tract infections as well as inconsistent evidence from previously reported randomized clinical trials (21–26). Our results suggest that routine double-J ureteral stent placement at the time of transplant may not be necessary in most patients; however, more prospective data is required in this matter.

In recent years minimally invasive operative techniques such as robot-assisted kidney transplantation (RAKT) has been successfully introduced in both living and deceased donor transplants (27–32). In one of these studies (29), a comparison of single (*N* = 127) vs. multiple (*N* = 21) vessel living-donor RAKT was performed in which the vascular reconstruction techniques performed on the back table were similar to ours reported here. In each of their cases, single arterial and venous anastomoses were performed after vascular reconstruction, and no unfavorable clinical outcomes for grafts with multiple vessels were found. Thus, both our data and this European study (29) reinforce the concept of the importance of performing careful on-bench extracorporeal vascular reconstruction of multiple vessels, allowing one single anastomosis to be performed in most cases during kidney transplantation, thereby, minimizing surgical risks.

Limitations of our study include the fact that this was a retrospective evaluation of consecutively transplanted patients performed at a single center by a single (although highly experienced) transplant surgeon. Second, while our overall cohort size of 210 patients was reasonable, sample sizes for certain subgroups of patients were relatively small. Third, while our overall results were excellent, with only 3.8% (8/210) of patients developing a post-operative (or surgical) complication post-transplant, statistical power to perform a multivariable analysis of this (primary) clinical outcome was limited due to the small number of events that occurred.

## Conclusions

Comparable results may be obtained in living-donor kidney transplantation when using allografts that require vascular reconstruction (on the back table), with no increased risks of developing DGF, post-operative (or surgical) complications, poorer renal function, or graft loss. Using the right kidney with a short renal vein was also not associated with any increased risk of developing a post-transplant vascular complication.

## Data Availability Statement

The raw data supporting the conclusions of this article will be made available by the authors, without undue reservation.

## Ethics Statement

The studies involving human participants were reviewed and approved by University of Miami Institutional Review Board. Written informed consent to participate in this study was provided by the participants' legal guardian/next of kin.

## Author Contributions

LG and GC: research design, data collection, and manuscript preparation. NP and LB: data collection. JG: statistical analysis and manuscript preparation. GG: research design. All authors contributed to the article and approved the submitted version.

## Conflict of Interest

The authors declare that the research was conducted in the absence of any commercial or financial relationships that could be construed as a potential conflict of interest.
